# A goodness-of-fit association test for whole genome sequencing data

**DOI:** 10.1186/1753-6561-8-S1-S51

**Published:** 2014-06-17

**Authors:** Li Yang, Jing Xuan, Zheyang Wu

**Affiliations:** 1 Department of Mathematical Sciences, Worcester Polytechnic Institute, 100 Institute Road, Worcester, MA 01609-2280, USA

## Abstract

Although many genetic factors have been successfully identified for human diseases in genome-wide association studies, genes discovered to date only account for a small proportion of overall genetic contributions to many complex traits. Association studies have difficulty in detecting the remaining true genetic variants that are either common variants with weak allelic effects, or rare variants that have strong allelic effects but are weakly associated at the population level. In this work, we applied a goodness-of-fit test for detecting sets of common and rare variants associated with quantitative or binary traits by using whole genome sequencing data. This test has been proved optimal for detecting weak and sparse signals in the literature, which fits the requirements for targeting the genetic components of missing heritability. Furthermore, this *p *value-combining method allows one to incorporate different data and/or research results for meta-analysis. The method was used to simultaneously analyse the whole genome sequencing and genome-wide association studies data of Genetic Analysis Workshop 18 for detecting true genetic variants. The results show that goodness-of-fit test is comparable or better than the influential sequence kernel association test in many cases.

## Background

According to the Catalog of Genome-Wide Association Studies updated by the National Human Genome Research Institute, approximately 7260 single-nucleotide polymorphisms (SNPs) have been identified for 770 traits in 1360 publications as of November 2012. However, researchers believe that a significant proportion of heritability of many complex traits is still missing [1,2]. The remaining genetic variants to be detected are either common variants with small allelic effects, or rare variants with relatively strong allelic effects. In both cases, the genetic effects are weak at the population level. Furthermore, only a small proportion of the avalanche of candidate variants are likely associated with a trait, which is a problem closely related to sparse signal discovery in statistics. It is very challenging to detect weak and sparse genetic effects via association.

To address this issue, we adopt a goodness-of-fit test (GOFT) [3] that has been proved to be optimal under a Gaussian means model [4]. That is, the boundary of the reliable detection of this method reaches the lowest possibility among all statistical tests when the signals are weak and sparse. Because the Gaussian means model is asymptotically equivalent to regression models [5], the GOFT is promising in detecting weak and sparse genetic effects through regression model fitting. In this work, we illustrate how to apply the test to whole genome sequencing (WGS) data by using the Genetic Analysis Workshop 18 (GAW18) data. The method is assessed under various rare variant collapsing strategies, and compared with the sequence kernel association test (SKAT) [6]. Moreover, because GOFT is a method combining *p *values, it has the potential to be used as a meta-analysis for incorporating data from different sources. We apply GOFT to simultaneously analyse WGS data and genome-wide association studies (GWAS) data for detecting genetic loci associated with systolic blood pressure (SBP). The results show that even without the sophisticated weighting scheme, GOFT is comparable to, and sometimes better than, SKAT under its best weighting scheme. In addition, at small *p *value cutoffs, the GOFT meta-analysis provides higher power than that when only WGS data is used.

## Methods

### Method 1: sequence kernel association test

Sequence kernel association test (SKAT) [6] is a supervised and flexible test for the associations between sets of genetic variants and a continuous or dichotomous trait. Through adjusting the variance of the random effect coefficients of the genetic variants, SKAT can consider different weights for different variants in contributing to the response trait. Typically, the rare variants are assigned with larger weights than the common variants based on the rare variant-common disease model [7]. We use the R package SKAT [8] for the WGS data analysis.

### Method 2: goodness-of-fit test

The problem of determining the associations between a set of genetic variants and the trait can be viewed as a multiple hypotheses testing problem. Under the null hypothesis that there is no genetic association, the *p *value from each genetic variant follows a uniform (0,1) distribution. So testing a group of variants can be considered as a GOFT that measures the consistency between the empirical distribution of the observed *p *values and the uniform distribution. Here we adopt a GOFT from Berk and Jones [3], which was proposed from large deviation theory, and then was proved optimal in detecting weak and sparse signals [4]. Let $p_{\left(1\right)}\leq \ldots \leq p_{\left(L\right)}$ be the sorted *p *values from *L *individual variants and the trait. The GOFT statistic is $G=L\cdot max_{1\leq j\leq \frac{L}{2}}K\left(\frac{j}{L},p_{\left(j\right)}\right)$, where,

(1) $$K\left(t,x\right)=\left[\begin{array}{c} tlog\left(\frac{t}{x}\right)+\left(1-t\right)\text{log}\left(\frac{1-t}{1-x}\right),\;\text{if}\;0<x<t<1,\\ 0,\;\text{if}\;0\leq t\leq x\leq 1,\\ +\infty ,\;\text{Otherwise}\text{.} \end{array}\right)$$

Comparing with many *p *value-based SNP-set testing methods that sum up all *p *values together in certain formulas [9], GOFT looks for the most representative *p *value to the SNP set. At the same time, unlike the minimal *p *value method that fixes the smallest *p *value to represent the set, GOFT adapts to the signal pattern through the maximization procedure. Such adaptation is critical because the *p *value of a true association is not necessarily the minimal value, especially when true associations are sparse and weak [4]. Another advantage is that the GOFT statistic only requires information from a set of *p *values to work, so it can be flexibly applied to different genetic studies based on the corresponding appropriate *p *values, or to meta-analyses that incorporate various data sources.

A permutation test can be applied to accommodate the various sizes of variant sets and the linkage disequilibrium structures among the variants. Specifically, let $G_{s}$ and $G_{sm}$, s=1,…,S,m=1,…,M, denote the GOFT statistics of the *s*^th ^genome segment window from the original data and from the *m*^th ^permutation of the genotype data, respectively. The empirical *p *value for the *s*^th ^window is $p_{s}=\#\left\{G_{sm}\geq G_{s},m=1,\ldots ,M\right\}/M$. The number of permutations *M *= 1000 was used in the following data analysis.

### Collapsing of rare variants

For the association study of complex diseases based on WGS data, a major challenge is to address rare variants that have weak statistical association as a result of small allele frequency. The GOFT is asymptotically optimal for weak and sparse signals, and is a right fit in this scenario. At the same time, because the effects of missense rare alleles are mostly in the same deleterious direction [10], collapsing the rare variant before GOFT is likely more efficient [11]. Furthermore, because common and rare variants contribute to complex diseases, it is good to combine information from both to facilitate the detection of associated genome segments. Following a literature work [12], we collapse rare single-nucleotide variants (SNVs) that allocate between adjacent common SNVs by summation of their genotype. Then the *p *values for associations of both collapsed rare variants and common variants are obtained and fed into GOFT test statistic in equation (1) to study the overall significance of variant groups.

## Results

For evaluating the above association tests, we used the WGS "dose" file of 1,215,399 SNVs and the GWAS file of 65,519 SNVs on chromosome 3 as the genotype data. The quantitative trait was the SBP for the 142 independent individuals who had no missing genotype. To assess how the SNV group's size may have affected the performance of these tests, we split chr3 into segments of fixed windows with 1 of 3 widths: 10 kilobase pairs (kbp), 100 kbp, and 500 kbp. Respectively at those 3 levels, the grouping strategy resulted in 19,472, 1950, and 391 windows, among which 87, 37, and 20 windows contained true SNVs that were either nonsynonymous or regulatory to SBP according to the GAW18 simulation [13]. The true windows and the 200 simulation replicates were used to evaluate power and type I error rate. We defined a SNV as a rare variant if it had a minor allele frequency less than 5%. The knowledge of the simulated true SNVs was only used for evaluating the power of the association tests, not for designing the tests and the data analysis strategies.

**Figure 1:**
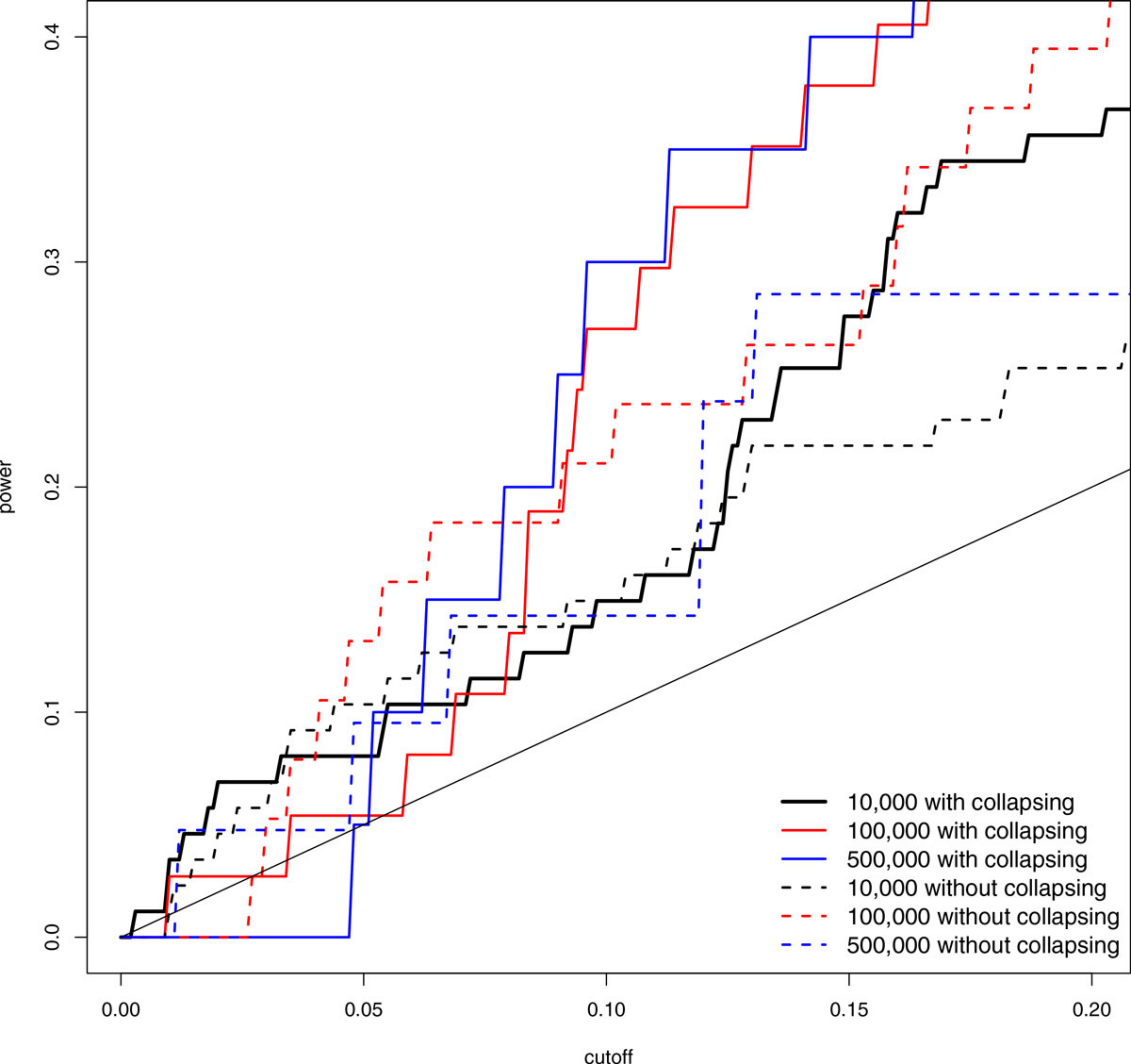
**Power of GOFT for different window sizes with or without collapsing variants**. Power is estimated by the true-positive rate of true-association windows on chr3 based on GAW18 simulation replicate 1.

For GOFT, we assessed its type I error rates as estimated based on the false-positive rate of the 19,385 false 10 kbp windows on chr3 over a sequence of cutoffs. The type I error rate was well controlled (results are available upon request). Figure 1 shows the assessment for the power of GOFT in detecting overall genetic associations, which was estimated by the true-positive rate of true-association windows based on GAW18 simulation replicate 1. We considered various window sizes with and without rare variant collapsing. Larger windows provided higher power at large cutoffs, but not at the small *p *values that are often used in practice. This is because large windows likely had more noise variants, which diluted the signals from true variants, and thus were harder to get very small *p *values. Meanwhile, rare variant collapsing did help to increase the power in general.

**Figure 2:**
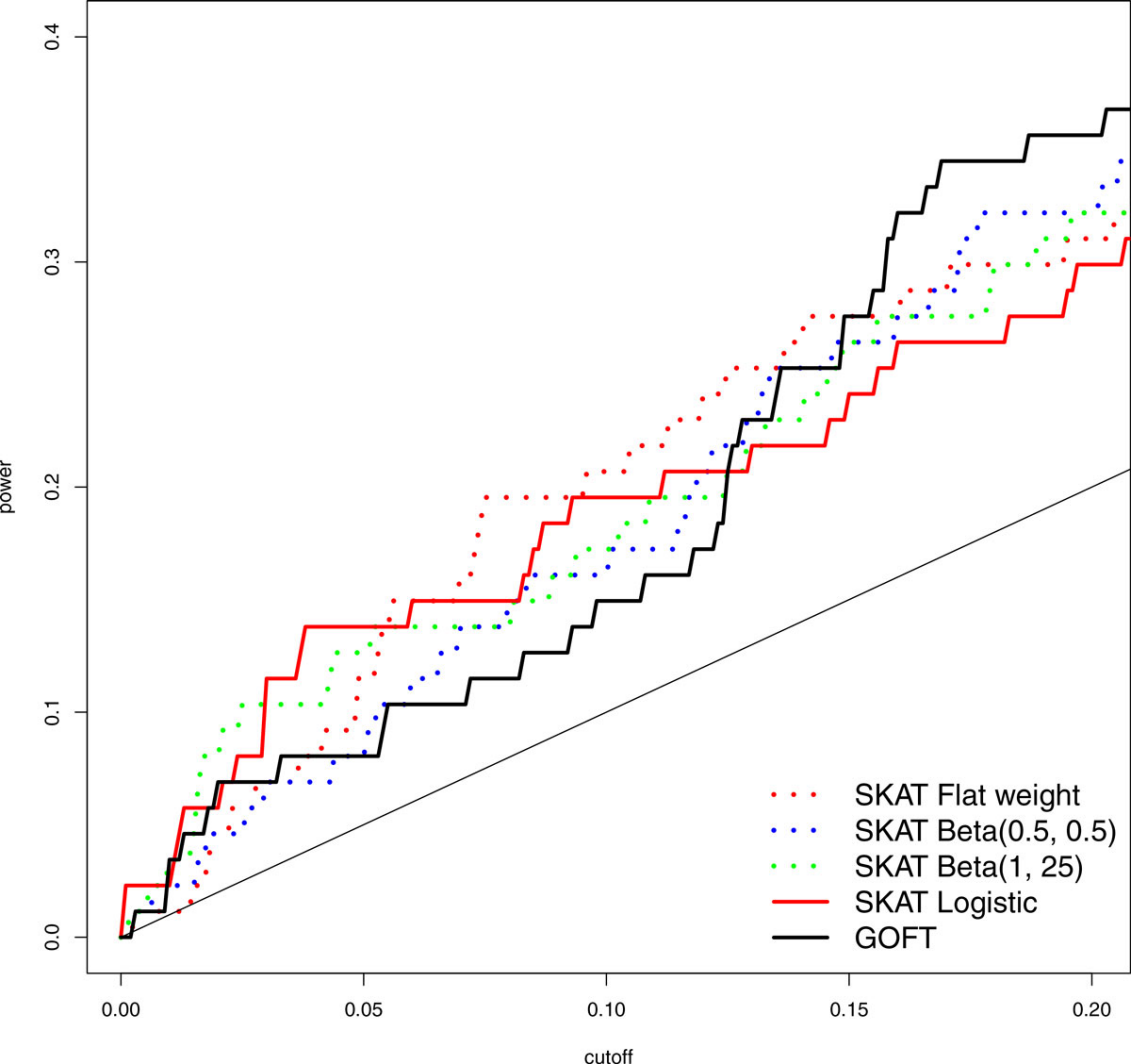
**Power of GOFT and SKAT under different weighting schemes**. Power is estimated by the true-positive rate of 87 true 10-kbp windows on chr3.

Under the window size of 10 kbp, we assessed SKAT with different strategies of weighting variants: flat weight, Beta(1, 1), Beta(0.5, 0.5), Beta(1, 25), and logistic(0.07, 150). Figure 2 shows the power of detecting the 87 true 10 kbp windows on chr3 over a variety of *p *value cutoffs. The Beta(1, 25) and logistic weights performed better for small *p *value cutoffs. Figure 2 also shows that GOFT was similar to the best SKAT setups for small *p *values. In fact, GOFTs had a larger area under the curve than SKATs when comparing their whole receiver operating characteristic (ROC) curves (results are available upon request).

**Figure 3:**
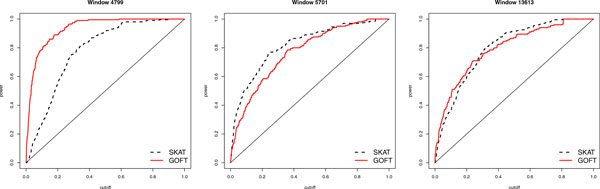
**Comparison patterns between GOFT and SKAT for detecting true windows**. *Left: *Window 4799 illustrates a case where GOFT is better. *Middle: *Window 5701 is an example where SKAT with logistic-weight is better. *Right: *Window 13613 is an example of both methods being similar.

To study the performance in detection of various patterns of genetic effects, we compared the power of GOFT and logistic-weight SKAT in detecting each of all 87 true 10-kbp windows on chr3. The power was estimated by the true-positive rate of a true window among 200 replicates. There are 3 patterns of comparisons: GOFT was better in 42 windows (Figure 3, *left panel*), SKAT was better in 27 windows (Figure 3*, middle panel*), and both were similar. Figure 3 illustrates examples of these comparisons based on the ROC curve (complete results are available upon request). GOFT seems better overall, but the comparison is not very significant (42 vs. 27, with a *p *value = 0.10). The type I error rate was well controlled here (results available upon request).

**Figure 4:**
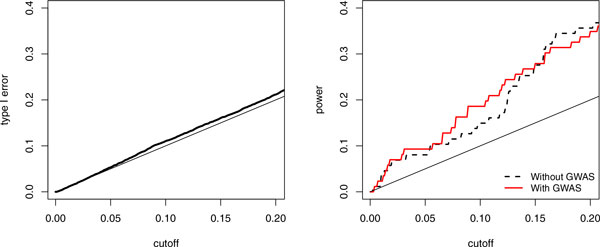
**Type I error rate and power for GWAS-WGS meta-analysis**. *Left: *Empirical type I error rate (ie, false-positive rate) in the meta-analysis. *Right: *Power of detecting the 87 true 10-kbp windows on chr3 when GWAS data were added or not.

Because GOFT only requires *p *values as the input, it has the potential to be used in a meta-analysis that incorporates data from different studies. Here we evaluated how much useful information the GWAS data could contribute to the WGS study. By mapping the "rs" IDs to the chromosome report from dbSNP, we calculated the *p *values of 65,519 GWAS SNVs on chr3. On average, 3.4 GWAS SNVs were added into each window (approximately a 5% increase). Figure 4 shows that the type I error rate control, after adding the GWAS SNVs, was still good (*left panel*) and that adding GWAS data helped to improve the power of GOFT in detecting true 10-kbp windows on chr3 in general.

## Discussion

We attempt to address the low power issue of association tests for WGS data from 2 aspects. First, we prefer to use tests specially designed for detecting weak and sparse genetic effects. For this purpose, the GOFT is asymptotically optimal in the sense that its asymptotic detection boundary is one of the lowest boundaries among all statistical methods. If signals are weaker or sparser than this boundary, no statistical methods would work well. Second, we try to borrow information from other data sets through meta-analysis. Although there has been some debate on how much of total heritability could be explained by GWAS data [7], the common agreement is that both common and rare variants contribute to complex diseases. It is potentially helpful to add GWAS data into WGS in order to increase the power. Our results show that both attempts are promising.

At the same time, several future works could be considered based on the limitations of the current study. First, the sample size is likely still small for either verifying asymptotical results or larger power of detecting weak genetic effects simulated in the data. It would be nice to further confirm the patterns of comparisons among these association methods by simulated and real data with much larger sample size. Second, gene-based collapsing can be applied as an alternative to the window scheme used here. Third, we have applied a simple rare variant collapsing process by direct summing the genotypes. This collapsing strategy is less sophisticated than the weighting strategy of SKAT. In fact, GOFT can further incorporate more successful collapsing strategies to improve its power, for example, by weighting the SNPs, like what SKAT and other methods have adopted [14-17]. Lastly, GOFT represents a first-stage analysis, which only seeks to answer where the associations are located; additional analyses could be required to determine the number and exact location of causal signals.

## Conclusions

We adopted a GOFT to WGS data analysis for detecting disease-associated genomic segments, and compared it to the SKAT by using the GAW18 simulation data with SBP_1 as response. Even without a sophisticated weighting scheme, in many cases, GOFT is comparable to or better than SKAT with the best weighting scheme. GOFT can be applied to a combination of GWAS and WGS data. Our results show that such meta-analysis has the potential to provide higher power over WGS data analysis only. In all cases, the power is still low for detecting overall heritability under the sample size of 142 independent individuals for genetic association study.

## Competing interests

The authors declare that they have no competing interests.

## Authors' contributions

ZW designed the overall study. LY, JX, and ZW conducted statistical analyses. LY and ZW drafted the manuscript. All authors read and approved the final manuscript.
